# Engineered endothelium provides angiogenic and paracrine stimulus to grafted human ovarian tissue

**DOI:** 10.1038/s41598-017-08491-z

**Published:** 2017-08-15

**Authors:** Limor Man, Laura Park, Richard Bodine, Michael Ginsberg, Nikica Zaninovic, Omar Alexander Man, Glenn Schattman, Zev Rosenwaks, Daylon James

**Affiliations:** 1 Center for Reproductive Medicine and Infertility, Weill Cornell Medical College, New York, NY 10065 United States; 2 Angiocrine Biosciences, Inc., San Diego, CA 92130 United States; 3 Tri-Institutional Stem Cell Derivation Laboratory, Weill Cornell Medical College, New York, NY 10065 United States

## Abstract

Despite major advances in tissue cryopreservation and auto-transplantation, reperfusion ischemia and hypoxia have been reported as major obstacles to successful recovery of the follicular pool within grafted ovarian tissue. We demonstrate a benefit to follicular survival and function in human ovarian tissue that is co-transplanted with exogenous endothelial cells (ExEC). ExECs were capable of forming functionally perfused vessels at the host/graft interface and increased both viability and follicular volume in ExEC-assisted grafts with resumption of antral follicle development in long-term grafts. ExECs that were engineered to constitutively express anti-mullerian hormone (AMH) induced a greater proportion of quiescent primordial follicles than control ExECs, indicating suppression of premature mobilization that has been noted in the context of ovarian tissue transplantation. These findings present a cell-based strategy that combines accelerated perfusion with direct paracrine delivery of a bioactive payload to transplanted ovarian tissue.

## Introduction

For patients diagnosed with cancer, survival rates are improving^1^, drawing increased attention to options for preserving reproductive options following remission. To protect their gametes from gonadotoxic therapies and defer reproductive options until disease remission, many patients are choosing to cryopreserve oocytes or embryos, a practice broadly referred to as “fertility preservation.” For pre-pubertal girls or women who require immediate chemotherapy, cryopreservation of oocytes and/or embryos is not an option. As an alternative, some patients opt to cryopreserve ovarian tissue and undergo auto-transplantation once in remission and ready to start a family. The frequency of positive outcomes of this approach is increasing^2–5^, yet graft survival and follicular output following auto-transplantation remain relatively low^6^ and despite numerous attempts to improve viability of ovarian cortical grafts using anti-oxidants^7, 8^, pro-angiogenic cytokines^9–12^, or mechanical manipulations^13^, graft ischemia in a 5 to 7 day window post-transplant remains a significant obstacle to maintaining tissue viability^14^.

Hypoxia and ischemia are critical determinants of survival post-transplant, but modulation of paracrine signaling also plays a large part in regulating follicular reserve^15, 16^. Anti-mullerian hormone (AMH), a member of the transforming growth factor beta (TGFβ) superfamily, was initially identified based on its role in promoting regression of Mullerian ducts during development of male sexual organs^17, 18^. But AMH is strongly expressed in growing follicles^19–21^ and the ovaries of AMH knockout mice display a “burnout” phenotype, with increased follicular mobilization and accelerated depletion of their primordial follicle stock^22^. These phenotypes suggest that AMH suppresses mobilization of primordial follicles, however, subsequent work in sheep concluded that AMH does not influence mobilization, but instead regulates the rate of early follicle progression^23^. AMH may perform multiple roles during follicular development, or subtle disparities in AMH function may exist between mono-ovulatory and poly-ovulatory species, but both suppression of follicular mobilization and slowing of early follicular growth rate would likely improve the long-term output of auto-transplanted ovarian tissue.

As graft resident endothelium is essential for recovery of tissue following xeno-transplantation^24^, one approach that may abbreviate the ischemic interval is supplementation of grafts with an exogenous source of ECs during transplantation. Moreover, stable integration of engineered cells could enable sustained delivery of therapeutic cytokines directly to the graft. Here, we have employed a cell-based strategy to both improve graft viability and provide a paracrine signaling impetus that can augment follicular reserve. This approach can provide a significant improvement in the output of functional oocytes for patients undergoing fertility preservation and the ExEC-based platform enables experimental interrogation of molecular regulators that have been implicated in follicular development.

## Results and Discussion

Due in large part to assisted reproductive technologies, cryopreservation protocols have significantly improved, yet a large degree of grafted tissue is still lost following *in vivo* transplant due to ischemia^14, 25–27^. Although a high degree of variability exists within the literature, and many papers describe substantial follicular reserve following transplantation, successful clinical attempts to restore fertility using heterotopic and orthotopic grafts typically require transplantation of large volumes of thawed ovarian tissue^28–30^, estimated to be as much as 55% of the entire ovary^4^.

**Figure 1:**
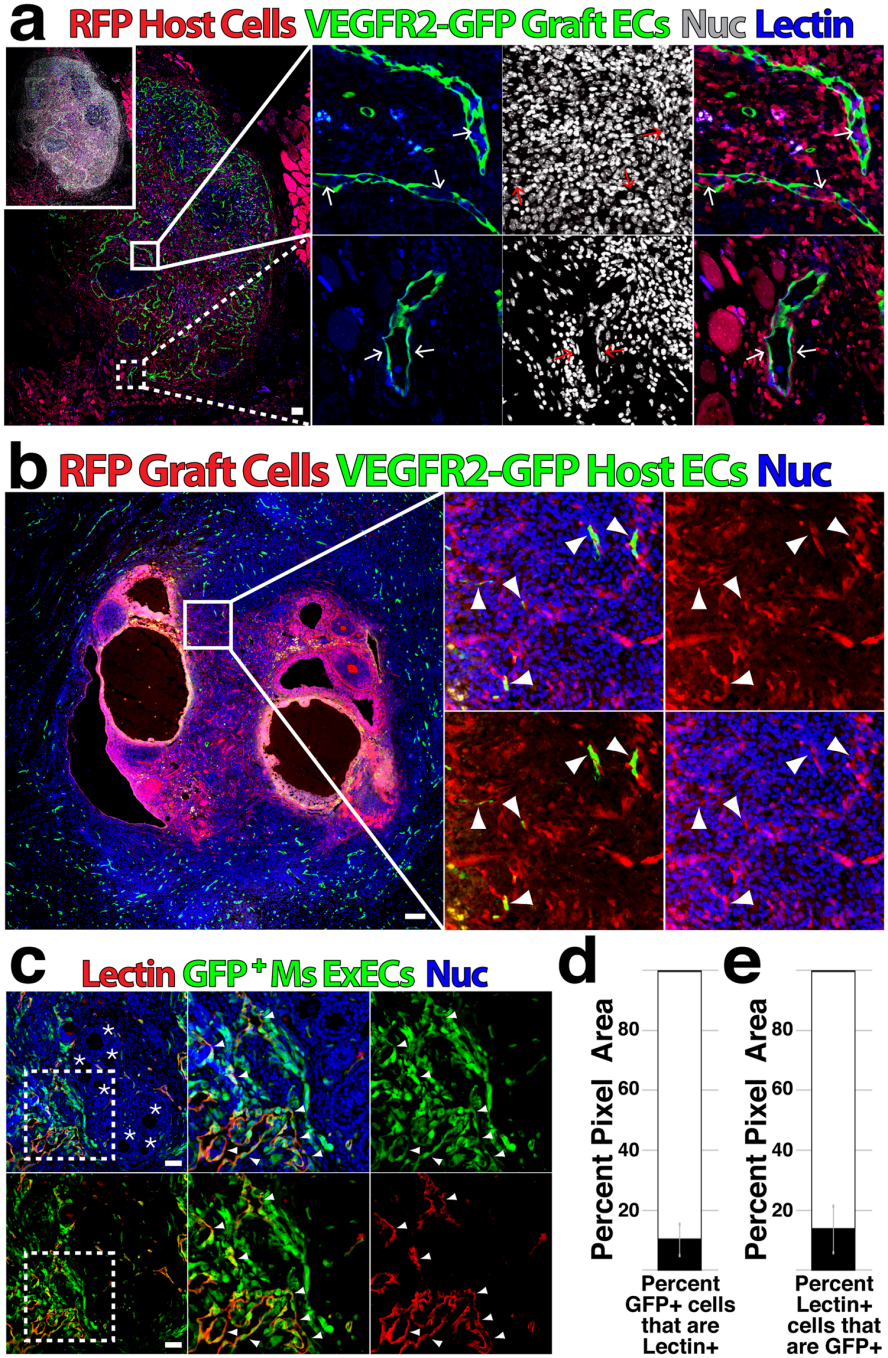
Host, graft and exogenous ECs contribute to revascularization of transplanted ovarian tissue. (**a**) Ovaries from VEGFR2-GFP mice were transplanted into RFP host; following two weeks, mice were injected with GIB4 lectin to label functional vessels and grafts were isolated to determine transit of host and graft ECs. (**b**) Ovaries from RFP mice were transplanted into VEGFR2-GFP host; following two weeks, grafts were isolated to assess infiltration of host vessels. (**c**) Ovaries were isolated from wild-type mice and co-transplanted with GFP^**+**^ ExECs derived from mouse heart; following two weeks mice were injected with GIB4 lectin to label functional vessels and grafts were isolated to assess contribution of ExECs to functional vessels. (**d**) Out of all GFP^**+**^ ExEC area, the percentage of Lectin^**+**^ area was measured. (**e**) Out of all Lectin^**+**^ area, the percentage of GFP^**+**^ ExEC area was measured. Insets in (**a**–**c**) are enlarged in the associated box. Error bars in (d and e) represent standard deviation between 10 representative tissue sections from 2 mice replicates. Scale bars = 100 μm.

Based on relatively low yields of oocytes obtained from clinically auto-transplanted tissue, we hypothesized that the lag in restoration of blood flow to the graft is a major detriment to viability. To define the relative contribution of host versus graft vessels to restoration of vascular perfusion, we performed syngeneic transplantation of ovaries between B6.Cg-Tg^**CAG-mRFP1**^ (RFP^31^) and Kdr^**tm2.1Jrt**^ (VEGFR2-GFP^32^) mice (Fig. 1a and b). After two weeks, an extensive degree of infiltration of host cells into the graft was evident, with RFP^**+**^ host cells combining with GFP^**+**^ graft-derived ECs to form functionally perfused chimeric vessels within the ovary and at the interface of graft and host (Fig. 1a). Similarly, GFP^**+**^ ECs derived from the host were observed within transplanted ovary in complex with graft derived RFP^**+**^ cells (Fig. 1b). To determine whether exogenous ECs (ExEC) were capable of contributing to functional vessels at the host/graft interface, we encapsulated wild-type mouse ovaries in plasma clots that were embedded with a suspension of E4ORF1-treated GFP^**+**^ mouse ECs^33^. Following two weeks, GFP^**+**^ ExECs were observed surrounding host tissue and contributed to functionally perfused vessels (Fig. 1c–e). Taken together, these data demonstrate substantial infiltration of graft tissue by host cells following transplant and affirm the potential for ExECs to contribute to functional vessels during this process.

**Figure 2:**
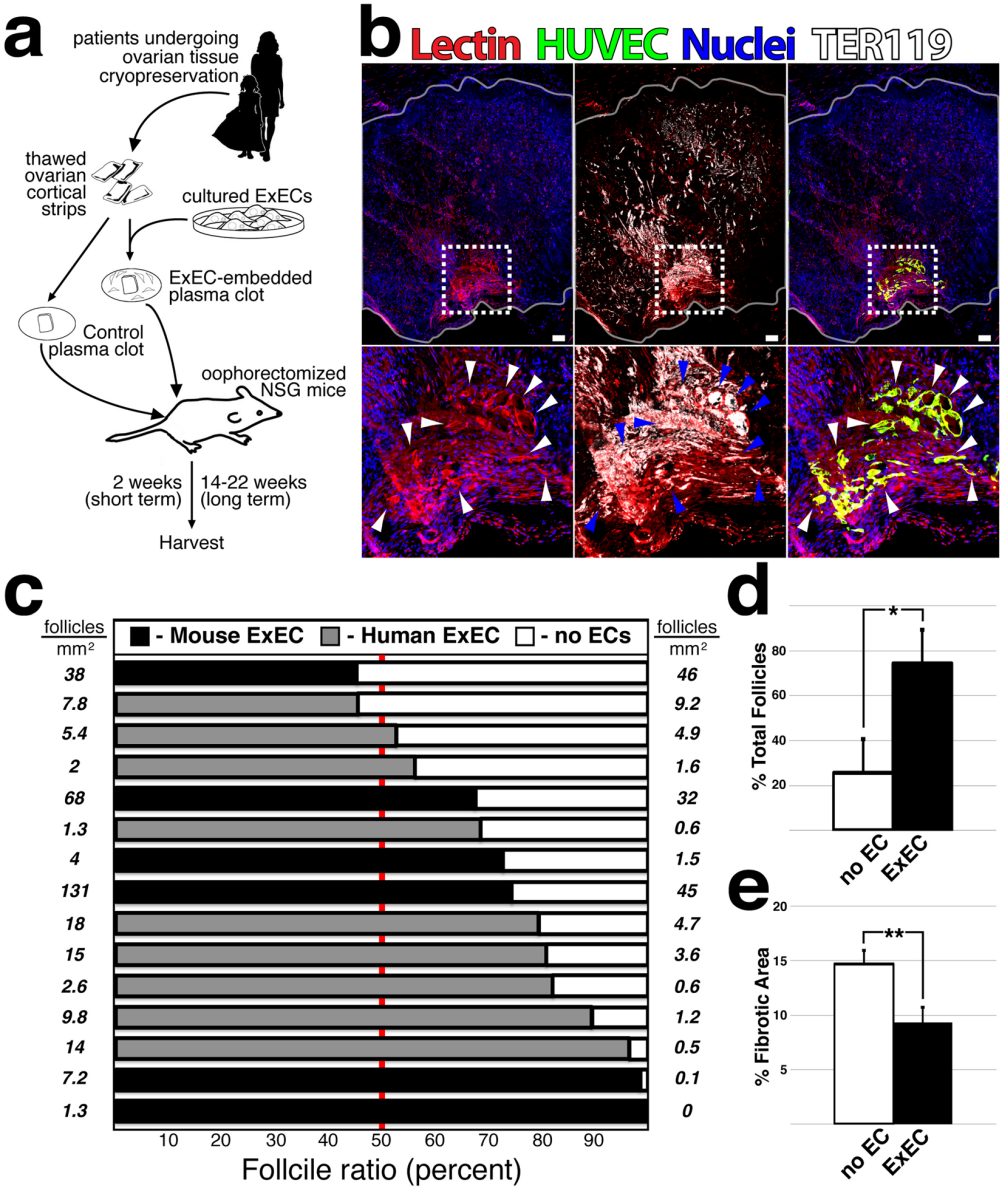
Co-transplantation of ovarian cortical strips with ExECs improves viability and preserves the follicular pool. (**a**) Scheme of the experimental design. Frozen-thawed human ovarian tissue was encapsulated in a plasma clot, with or without endothelial cells; ExEC-embedded plasma clot, Control plasma clot, respectively. The clots were transplanted into oophorectomized NSG mice and harvested at the end point of the experiment. (**b**) Human cortical grafts co-transplanted with hUVEC-derived ExECs (green); red blood cells are labeled by TER-119 and boundaries of the graft are outlined in white. (**c**) The ratio of surviving normal follicles in human ovarian tissue xenografted with and without the mouse or human ExECs in comparison to no cells. Each row represents a replicate originating from the same patient transplanted into the same mouse, serving as its own control. On each side of the graph, the density of the follicles per millimeter squared is indicated. (**d**) Median percentage of total follicles from transplantation with and without mouse or human ExECs + MAD. *P < 0.05. (**e**) Median percentage of the fibrotic area from transplantation with and without mouse or human ExECs + MAD. **P < 0.001.

To determine whether co-transplantation of ExECs provides a benefit to patient tissue, we utilized tissue that had been frozen and thawed to optimize retention of follicular reserve (Supplementary Figure 1a). We divided thawed cortical strips into equal sized pieces, embedded them in a plasma clot with or without ExECs, and performed bilateral engraftment into immuno-compromised (NSG) mice (Fig. 2a). After two weeks GFP-labeled ExECs formed functionally perfused vessels at the interface of graft and host tissue, thereby restoring blood flow to tissue (Fig. 2b and Supplementary Figure 1b). Notably, early experiments also included human foreskin fibroblasts as a non-vascular cell control, however these grafts showed poor tissue quality following recovery (Supplementary Figure 1c), and this condition was excluded from further study. Quantification of follicular survival at 2 weeks following transplant demonstrated a significant benefit to relative follicle count in ExEC-assisted grafts (Fig. 2c and d and Table 1), with a majority of experiments favoring ExECs derived from either mouse (heart ECs) or human (hUVEC). Interestingly, follicular volume was globally improved in smaller sized fragments, however the benefit of ExECs to follicular survival was more robust for larger fragments (Supplementary Figure 1d). Trichrome staining of graft sections, which has previously been used to assess bovine ovarian tissue in mouse xenografts, revealed reduced fibrotic area in ExEC-assisted grafts (Fig. 2e), suggesting that improved tissue viability accounted for the increase in follicular volume.

**Table 1:** List of patient characteristics for ovarian tissue fragments that were xenografted into NSG mice.

Patient #	Patients’ age at cryopreservation	Fresh-F/Thawed- T	Main reason for cryopreservation	Chemo Rx	# of pieces used	# of mice used
1	19	F	Hodgkin’s	ABVD	6	1
			Lymphoma			
2	32	F	Hodgkin’s	Yes	6	3
			Lymphoma			
3		T		No	6	2
4		T		No	4	2
5	19	T	Ewing’s sarcoma	No	12	6
6	17	T	Hodgkin’s stage IV	No	2	1
7	5	T	Beta Thalassemia	No	6	3
8	6	T	Thalassemia Major		2	1
9	17	T	Cancer	No	4	2
10	26	T	Hodgkin’s	ABVD,	8	4
			Lymphoma	Lupron depot		
11	18	T	Lymphoblastic lymphoma	Doxorubicin,	4	2
				Ara C, vincristine, PEG-asparaginase, MTX		
12	5	T	Beta thalassemia	No	4	2
13	18	T		Chemo	8	4
14	18	T	Non-Hodgkin Lymphoma		4	2

**Figure 3:**
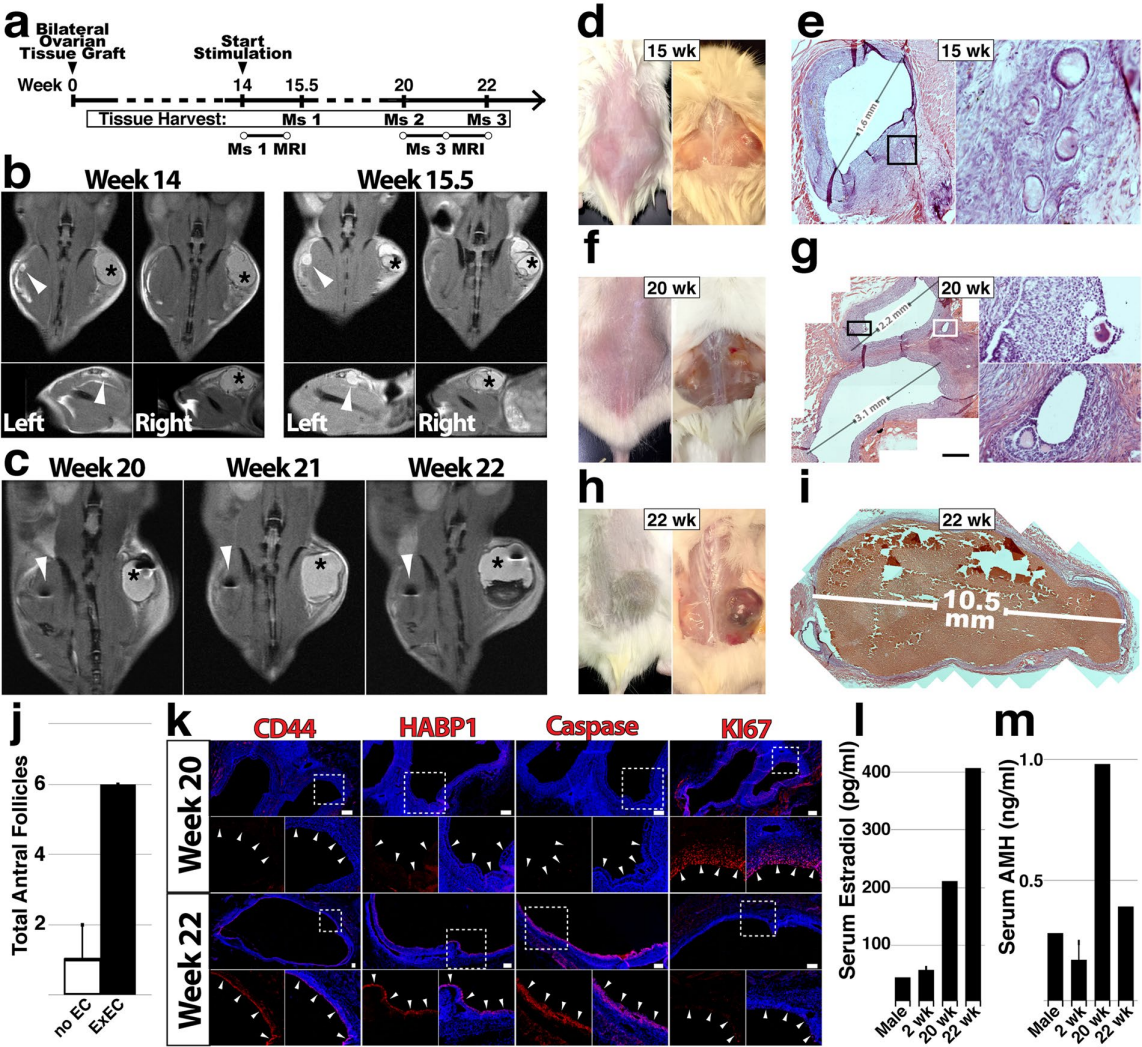
Long-term viability and function of ovarian tissue grafts are improved by ExECs. (**a**) Experimental scheme for long-term xenograft of ovarian cortical tissue. (**b**) Mouse #1 was xenografted with tissue from a 6-year-old donor and monitored by MRI at the onset of stimulation (14 weeks, left) and again after ten days of stimulation (right). (**c**) Mouse #3 was xenografted with tissue from a 19-year-old donor and monitored by MRI at 6 (20 weeks post-transplant), 7 (21 w) and 8 (22 w) weeks following the onset of stimulation. d and e) Mouse #1 was sacrificed after 15.5 weeks and control and ExEC-assisted grafts were harvested for histological analysis; in (**d**) the graft on the right side is the ExEC-assisted graft and a histological view of the control graft is shown in (**e**). (**f**,**g**) Mouse #2 was sacrificed after 20 weeks and control and ExEC-assisted grafts were harvested for histological analysis; the ExEC-assisted graft is shown in (**g**). (**h**,**i**) Mouse #3 was sacrificed after 22 weeks and control and ExEC-assisted grafts were harvested for histological analysis; the ExEC-assisted graft is shown in (**i**). (**j**) Median of total number of antral follicles in ExEC-assisted versus control grafts from Mice 1–3 + MAD. N = 3. k) Sections of the ExEC-assisted grafts from Mouse #2 and Mouse #3 were stained for molecular markers shown. (**l**,**m**) Serum isolated from control (male), short-term-2 weeks and long-term-20,22 weeks xenografted mice was tested by ELISA for levels of human Estradiol (**l**) and AMH (**m**). Insets in (**e**,**g**,**k**) are enlarged in the associated box. Scale bars = 100 μm.

To assess the long-term survival and function of ovarian tissue grafts, we performed bilateral transplant of patient tissue with and without hUVEC-derived ExECs (Fig. 3a). Following 14 weeks, mice were stimulated daily with menotropins for variable lengths of time before animals were sacrificed, and two mice were monitored by MRI to assess the presence/growth of follicles over time (Fig. 3b and c and Supplementary Videos 1–3). Developing follicles were noted in both control (Fig. 3e) and ExEC-assisted (Fig. 3g and i) grafts, with more and larger sized follicles in the ExEC-assisted grafts. Indeed, two control grafts (20 and 22 weeks) contained 0 and 1 antral follicles, compared to 6 and 5 antral follicles in corresponding ExEC-assisted grafts (Fig. 3c and f–j). Comparison of tissue from the same patient that was stimulated for 6 or 8 weeks indicated ovulatory potential of grafts. Longer stimulation resulted in a large (>10 mm) follicle in the ExEC-assisted graft (Fig. 3i), which displayed increased positivity for ovulatory markers CD44^34^ and HABP1^35^, increased apoptosis (Caspase), and reduced KI67^**+**^ proliferative cells (Fig. 3k). Collectively, long term-grafts with ExECs showed a qualitative (Fig. 3b–i and k) and quantitative (Fig. 3j) advantage relative to control, and levels of human Estradiol (Fig. 3l) and AMH (Fig. 3m) were increased in host sera.

**Figure 4:**
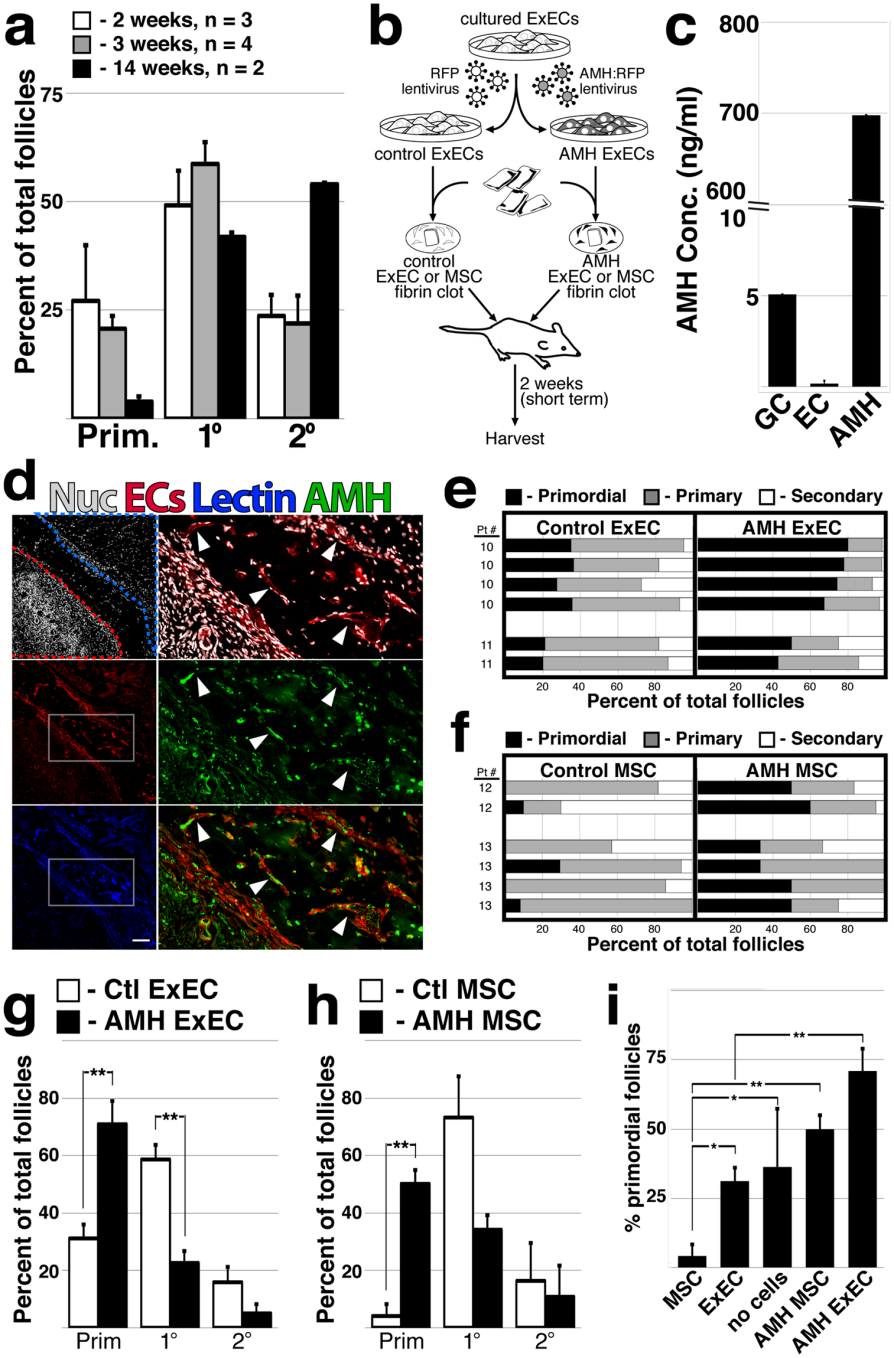
ExECs engineered to express AMH preserve a quiescent follicular pool. (**a**) The proportion of follicles was quantified for multiple ovarian tissue fragments from the same patient that were xoengrafted for long and short term intervals; n = 3 at 2 weeks, n = 4 at 3 weeks and n = 2 at 14 weeks. (**b**) Scheme of the experimental design. Frozen-thawed human ovarian tissue was encapsulated in a fibrin clot, with either ExECs/MSCs transduced with an RFP lentiviral particle serving as a control, or ExECs/MSCs transduced with an AMH-RFP lentiviral particle generating AMH-ECs/MSCs. The clots were transplanted into oophorectomized NSG mice and harvested at 2 weeks. (**c**) ExECs were transduced with lentivirus encoding secreted human AMH; cell culture supernatant of AMH-transduced ExECs was compared to COV-434 culture granulosa cell tumor line and control ExECs. (**d**) Two weeks after transplant, ovarian tissue fragments that were co-transplanted with AMH-ExECs were stained with an antibody specific for AMH protein. (**e**) The relative proportion of follicles in xenografts co-transplanted with control and AMH-ExECs was quantified after 2 weeks (n = 6). (**f**) The relative proportion of follicles in xenografts co-transplanted with control and AMH-MSCs was quantified after 2 weeks (n = 6). (**g**) The median + MAD of the relative proportion of follicles was compared in xenografts transplanted with control and AMH-transduced ExECs (n = 6) following 2 weeks. (**h**) The median + MAD relative proportion of follicles was compared in xenografts transplanted with control and AMH-transduced MSCs (n = 6) following 2 weeks. (**i**) The percentage of the observed primordial follicles per graft in xenografts transplanted with MSCs (n = 6), ExECs (n = 6), AMH-MSCs (n = 6), and AMH-ExECs (n = 6) was compared in aggregate to control conditions (no cells, n = 15). Insets in (**d**) are enlarged in the boxes to the right; red and blue stroke lines in (**d**) outline ovarian tissue and host tissue, respectively. Error bars in (**c**) represent standard deviation between 3 replicates. Error bars in (**a**,**g**–**i**) represent MAD between number of replicates listed or shown in the graph. Scale bar in (**d**) = 100 μm. *P < 0.05, **P < 0.005.

Disruption of physiological signaling mechanisms that regulate follicular activation may also play a role in undermining graft output. Numerous studies have noted premature global activation of the follicular pool in the context of transplantation of cryopreserved human follicles^36^ and ovarian tissue^37–39^. Ovarian tissue transplanted in our study retained an average of less than 30% of follicles in the primordial stage after two weeks (data not shown). Indeed multiple grafts from a single patient at 2, 3 and 14 weeks revealed a shift in the follicular pool away from quiescence with increasing graft duration (Fig. 4a). Global activation of the follicular pool is especially deleterious because it occurs during the ischemic window when increased metabolic demands of growing follicles cannot be sustained. Because AMH has been suggested as a physiological factor that exerts a repressive input on activation and/or growth of follicles^22, 23^, we used lentivirus to engineer ExECs that constitutively express and secrete AMH (Fig. 4b). Compared to granulosa cell tumor cells, which secreted a basal level of AMH in cell culture supernatant, non-transduced ExECs expressed undetectable amounts of AMH, but levels were increased more that 100-fold above GCs following lentiviral transduction (Fig. 4c). Two weeks after co-transplantation, vessels derived from AMH-ExECs were observed at the host-graft interface and immunostaining revealed abundant AMH protein in the lumen of these vessels (Fig. 4d); co-transplantation with AMH-ExECs resulted in an approximately two-fold increase in the percentage of primordial follicles relative to control ExECs, while the percentage of primary follicles was decreased (Fig. 4e and g). In order to test the function of AMH independently from the pro-angiogenic influence of ExECs, we co-transplanted tissue with control and engineered mesenchymal stem cells (MSC) (Fig. 4f and h). Although control MSCs generally induced a greater proportion of growing follicles relative to ExECs, AMH-MSCs increased retention of primordial follicles by 10-fold relative to control MSCs. Indeed, comparison of MSCs, ExECs, non-cellular grafts (Ctl), AMH-MSCs ExEC and AMH-ExECs revealed the most significant benefit to the retention of the quiescent follicular pool conferred by AMH-ECs (Fig. 4i).

Here we demonstrate that co-transplantation of ExECs can provide a significant benefit to ovarian tissue viability and retention of follicular reserve following xenograft. Fertility preservation via freezing of ovarian tissue is gaining traction, with calls to move auto-transplantation past experimental status to broad clinical application^3, 5^ and increased focus being placed on referral of newly diagnosed cancer patients to fertility specialists^40, 41^. Pre-pubertal patients stand to benefit considerably from mainstream adoption of ovarian cryopreservation^42, 43^, as their follicular reserve is high, however the increased density of primordial follicles at this stage is balanced by the small size of ovaries. As such, suppression of molecular inputs that drive premature mobilization of the follicular cohort is vital to the longevity and productivity of these grafts. Reinforcement of signals that regulate follicular homeostasis in steady state ovaries, combined with reduced ischemia/hypoxia, could improve the output and longevity of grafts, preserving reproductive as well as endocrine function for a sustained period of time. However, further experiments in animal models that fertilize and transfer oocytes to assess oocyte/embryo quality would be an important pre-requisite to clinical application.

Despite its standard use as a diagnostic measure of ovarian reserve and numerous studies interrogating its role during follicular development, the function of AMH in the human ovary remains unclear. Other TGFβ superfamily signaling components have also been implicated in follicular mobilization – spontaneously derived mutant strains of ewes that exhibit increased ovulation rate and multiple births have a mutation in the type-IB BMP receptor BMPR1B^44^; a range of similar phenotypes has been traced to mutation or immunological depletion of the secreted factor BMP15^45, 46^ or its paralog GDF9^45^. While mouse knockout studies suggest that AMH suppresses mobilization of primordial follicles^22^, studies in mono-ovulatory species (sheep) suggest that AMH regulates the growth rate of early follicles^23^. The capacity for AMH ExECs to shift the balance of the follicular cohort toward a primordial phenotype (Fig. 4) suggests that AMH is suppressing follicular activation in this case, however our study lacks an evaluation of long-term grafts that could interrogate the function of AMH during follicular growth. Regardless of the precise mechanisms of AMH function during physiological folliculogenesis, our approach provides a novel means of tempering premature mobilization of follicular reserve via direct paracrine delivery of bioactive cytokines at the margins of grafted ovarian tissue.

## Materials and Methods

### Mouse RFP to VEGFR2-GFP ovarian grafts and vice versa

All procedures were approved and experiments were performed in accordance with the guidelines and regulations of the Institutional Animal Care and Use Committee (IACUC) of Weill Cornell Medical College. VEGFR2GFP females aged 9–16 weeks (n = 2) were obtained from Jackson Laboratories, USA, and housed under specific pathogen–free conditions. Mice were oophorectomized and two weeks later transplanted with a whole ovary from an age matched B6.Cg-Tg(CAG-mRFP1^31^) mouse. Grafts were collected at 2 weeks after transplantation. The same procedure was performed using B6.Cg-Tg(CAG-mRFP1) females as host for transplant of B6 VEGFR2-GFP^32^ ovaries.

### Co-transplantation of WT ovary into WT mouse

C57/B6 female mice aged 10–16 weeks (n = 2) were obtained from Jackson Laboratories, USA, and housed under specific pathogen–free conditions. Two weeks after oophorectomy, whole ovary from age-matched mice were embedded in a plasma clot along with GFP-labeled mouse ECs and transplanted into the gluteus muscle of C57/B6 mice. Grafts were collected at 2 weeks post-transplantation and analyzed histologically.

### Collection of Human Ovarian Tissue

The tissue was collected from patients scheduled for chemotherapy or radiotherapy for cancer treatment or prior bone marrow transplantation. The institutional review board (IRB) Committee of Weill Cornell Medical College approved the collection of tissue for potential autologous use and upon the patients’ informed consent a donation of up to 10% of their ovarian tissue for research use was performed. Samples from 14 patients were selected for this study. All identifying information was removed from samples before commencement of the study, except age and basic disease condition treatment (see Table 1) as listed in the manuscript. All xenotransplant experiments using fresh or thawed ovarian tissue were performed in accordance with relevant guidelines and regulations.

### Ovarian Tissue Slow Freezing and Rapid Thawing

Ovarian tissue freezing/thawing was performed as previously described^47^. Briefly, ovarian tissue was isolated and dissected, the medulla was removed the cortical tissue was cut into slivers between 1–1.5 mm in thickness. Cortical strips were transferred to cryogenic vials containing DMSO as a cryoprotectant and cooled in a programmable freezer at 2 °C/minute to −7 °C, and then at 0.3 °C/minute until sample temperature reached −40 °C, then cooling at a faster rate of 10 °C/minute to −140 °C. Tubes were then immersed and stored in liquid nitrogen (−196 °C). When required, Vials were thawed rapidly, at ~100 °C/min by immersing in a 30 °C water bath, then the cryoprotectant was gradually removed using lower concentrations of DMSO in a stepwise manner, with gentle agitation for 5 min in each step. The thawed tissue was kept on ice in fresh medium until the transplantation was performed.

### Transplantation to NSG Mice

Thirty-five 10–14-week-old female NSG^48^ mice (Jackson Labs) were used. From each human ovarian tissue sample, we were able to transplant up to 6 mice, bilaterally with an average of 2.63 ± 1.08 mice being transplanted with 5.42 ± 2.65 cortical ovarian pieces per patient sample. As previously shown^49^, the density of primordial follicles varied in cortical fragments within the ovary and between ovaries and samples lacked homogeneous follicular distribution^50^, with follicles tending to be located in clusters and not evenly distributed throughout the tissue^51^. On this basis, we considered each piece as a replicate by itself. Mice were anesthetized by using 150 mg/Kg Ketamine, 15 mg/kg Xylazine IP. After medial dorsal incision, they were bilaterally oophorectomized and 1–3 pieces of fresh or thawed human ovarian tissue (sized: 1 × 2 mm–6 × 3 mm surface dimensions), encapsulated in a plasma clot containing single cell suspension was co-transplanted under the fascia of the Gluteus Maximus on one side and a similar sized piece of ovarian tissue without ECs on the other. In later experiments the tissue was co-transplanted with lentiviral transduced compared to non-transduced ECs, encapsulated in a fibrin clot in the same anatomical location. The fascia was sutured with one or two stitches (6/0 polysorb) and the dorsal wall was then closed using 4/0 biosyn. After closure of the skin the mice were treated with sub-cutaneous (S/C) Lidocaine 0.5% (1.2 ml/Kg) at the incision site, and S/C Buprenorphine (1 mg/Kg) was administered as well. The animals were kept in sterile conditions until the end point of the experiment. Engrafted tissue was harvested at different time points, as indicated in each one of the experimental designs.

### Encapsulation of the ovarian tissue

The tissue was encapsulated in a plasma clot, and in later experiments, AMH-ECs vs ECs and AMH-MSCs vs MSCs, in fibrin clots. Preparation of a plasma clot: whole blood was drawn into 5-mL sodium citrate-coated sterile tubes and centrifuged at 3000 rpm for 15 min. The supernatant was recovered and frozen until use. When needed, a vial of plasma was thawed and placed on top of the ovarian piece, for the ECs Tx it was re-suspended with a suspension of single cells. Clotting was induced by adding 2 μl of 1 M CaCl2, followed by incubation at 37 °C for 30 min. The same procedure was performed when creating the control clot, which was prepared without addition of cells. Fibrin was prepared from Bovine Fibrinogen (Sigma-Aldrich- F8630) in a final concentration of 10 mg/ml, and Thrombin from human plasma (Sigma-Aldrich- T1063) at a final concentration of 5 U/ml. A droplet of 70 μl containing a mixture of Fibrinogen, cells, medium and Thrombin was placed on top of the ovarian fragment. Clotting was induced by adding Thrombin, followed by incubation at 37 °C for 30 minutes. Cells were added at a concentration of 20,000 cells per 1 mm^2^.

### Gonadotropin Stimulation

Mice harboring long-term grafts (15, 20 and 22 weeks) were treated with menotropins (Menopur®, Ferring) once daily (2 IU/day). It was injected S/C or intramuscularly (IM) starting after 14 weeks of engraftment. Mice were stimulated for a range of 10 days, in the 15 weeks to eight weeks, in the 22 weeks.

### Histology

Grafts were fixed with 4% paraformaldehyde (PFA) and dehydrated with sucrose 35%, Optimal Cutting Temperature (OCT) - embedded and serially sectioned (10-μm sections). H&E staining was used for differential follicle counts; follicle counts were conducted on every third section (10 μm) of entire ovarian tissue. All ovarian follicles in the section were counted and classified according to stage into primordial, primary, secondary, and antral follicles, following specific criteria^52^. Primordial follicles were characterized by one oocyte surrounded by a single layer of flattened pregranulosa cells (GC). Intermediate follicles were counted as primary, the oocyte was surrounded by a single layer of both flattened and cuboidal GCs. Primary and secondary follicles contained an oocyte surrounded, respectively, by one single layer and at least two layers of cuboidal GCs, but no antrum. Antral follicles showed the development of an antral cavity. Only normal morphologic follicles were counted. In order not to count the same antral follicle twice, only mid-section containing the oocyte was counted. Follicular density was thus calculated to obtain the number of ovarian follicles per unit area (square millimeters). Sample sections were used for immunohistochemistry and stained for apoptosis with Rabbit anti-Human Cleaved Caspase-8 mAb (1:100 dilution; from Cell Signaling Technology). Proliferation staining was conducted with Rabbit anti-Human Ki67 antibody (1:100 dilution; from Abcam). Other antibodies used for immunohistochemistry were Rabbit anti-Human Cytochrome P450 1B1 Ab (1:200 dilution; from Thermo-Fisher scientific), Rat anti-mouse Alexa fluor 488 TER-119 (1:100 dilution; from BioLegend), Rabbit anti-Human C1QBP(HABP1) (1:200 dilution; from Cell signaling Technology), FITC anti-Human CD44 (1:50 dilution; from BioLegend), and Rabbit anti-AMH Ab (1:50 dilution; from Thermo-Fisher scientific). All secondary antibodies were from Invitrogen (life technologies) and were used in a 1:1000 dilution.

Trichrome staining, for the evaluation of the fibrotic areas, was performed by using a kit from Sigma-Aldrich. From each transplanted graft, 3 sections were stained. In order to represent the entire graft, we analyzed a middle section and a 600-μm depth section from the upper and lower sides of the graft. Stained sections were scanned and analyzed using Image J to quantify the surface area. The surface area was manually outlined and the relative percentage out of the entire graft area was calculated (Image J software). Each graft was represented as the average of the 3 sections that were analyzed. For labeling functional blood vessels we used Isolectin GS-IB4 From Griffonia simplicifolia, Alexa Fluor® 647 Conjugate (Thermofisher) in a final concentration of 0.5 mg/ml. A few minutes prior to harvesting the tissue the lectin was injected intravitally, while mice were anesthetized with isoflurane.

### Statistical Analysis

Data from treated and untreated groups were compared by non-parametrical test Mann Whitney U with significance defined as P ≤ 0.05. Data from grafts treated with different cell types were compared using Kruskal Wallis. Significance was defined as P ≤ 0.05.

### Blood plasma measurements

Blood was extracted from mouse heart at time of death and separated by centrifugation; at 1300 g for 10 minutes, then the plasma was frozen at −20 °C. Plasma AMH concentration was quantified with an AMH ELISA (enzyme-linked immunosorbent assay) kit (Becton Dickenson). Estradiol levels were measured by IMMULITE (Siemens).

### Cells Supernatant Measurements

Supernatants were collected after 48 hours of culture and quantified for AMH levels using IMMULITE (Siemens).

### Endothelial cell origin

For co-transplantation we used exogenous ECs modified by the adenoviral gene fragment E4-ORF1 (E-CEL^**TM**^, Angiocrine Bioscience), which preserves the angiogenic profile of ECs *ex vivo*. Mouse ECs were originally isolated from heart; human ECs were originally isolated from neonatal umbilical vein (hUVEC).

### Neonatal Human Foreskin Fibroblast Origin

Cells were purchased from GlobalStem, named NUFF-1, passage -9. Transplantation method was the same as for ECs.

### Granulosa Cell origin

The line of immortalized Granulosa cells (COV434) was purchased from Sigma-Aldrich.

### Isolation of MSCs

Fragments of ovarian medulla were collected while processing ovaries for freezing. The tissue was minced and incubated with Dispase (GIBCO) in basal medium. Following centrifugation the supernatant was discarded and the pellet was re-suspended in fresh basal medium. The suspension was passed through a 100 μm cell strainer, and centrifuged again. The pellet was re-suspended in culturing medium (DMEM-low glucose-1 g/L, 10% FBS, 1% L-glutamine, 1% NEAA, 1% pen/strep.)

### Lentiviral Vectors and Transduction of Cells

Lentiviral particles (GeneCopoeia) were added to cultured ECs and incubated for 48 hr.

### Data availability

The datasets generated during and/or analyzed during the current study are available from the corresponding author on reasonable request.

## Electronic supplementary material

Supplementary Material

Supplementary Video 1

Supplementary Video 2

Supplementary Video 3
