# Supplementary material for: Engineered endothelium provides angiogenic and paracrine stimulus to grafted human ovarian tissue
Source: Sci Rep. 2017 Aug 15;7:8203. doi: 10.1038/s41598-017-08491-z (PMC5557862; doi:10.1038/s41598-017-08491-z)

Supplementary Material

Engineered endothelium provides angiogenic and paracrine stimulus to grafted human ovarian tissue

Limor Man[1], Laura Park[1], Richard Bodine[1], Michael Ginsberg[2], Nikica Zaninovic[1,3], Omar Alexander Man[1], Glenn Schattman[1], Zev Rosenwaks[1,3] and Daylon James[1,3] *

[1] Center for Reproductive Medicine and Infertility, Weill Cornell Medical College, New York, NY, 10065.

[2] Angiocrine Biosciences, Inc., 1300 York Avenue, New York, NY, 10028

[3] Tri-Institutional Stem Cell Derivation Laboratory, Weill Cornell Medical College, New York, NY, 10065

Supplementary Figure 1. a) Following thaw of cryopreserved ovarian cortical strips, cytochrome P450 labeled follicles (red) are present in abundance. Insets are enlarged in the associated box. Scale bars = 100 mm. b) Xenograft of human ovarian tissue co-transplanted with ExECs (green) and intra-vitally administered GIB4 lectin (red). c) Representative sections of ovarian grafts co-transplanted with ExECs, without cells and with human foreskin fibroblasts. Insets are enlarged in the associated box. d) Median density +MAD of total follicles per squared millimeter in smaller (3-6 mm^2, n=7) or larger (8-18mm^2, n=8) ovarian grafts with and without ExECs.

Supplementary Video 1. Mouse #1 MRI showing coronal sections at 14 and 15 weeks.

Supplementary Video 2. Mouse #1 MRI showing sagittal sections at 14 and 15 weeks.

Supplementary Video 3. Mouse #3 MRI showing coronal sections at 20, 21 and 22 weeks.

Supplementary Figure 1

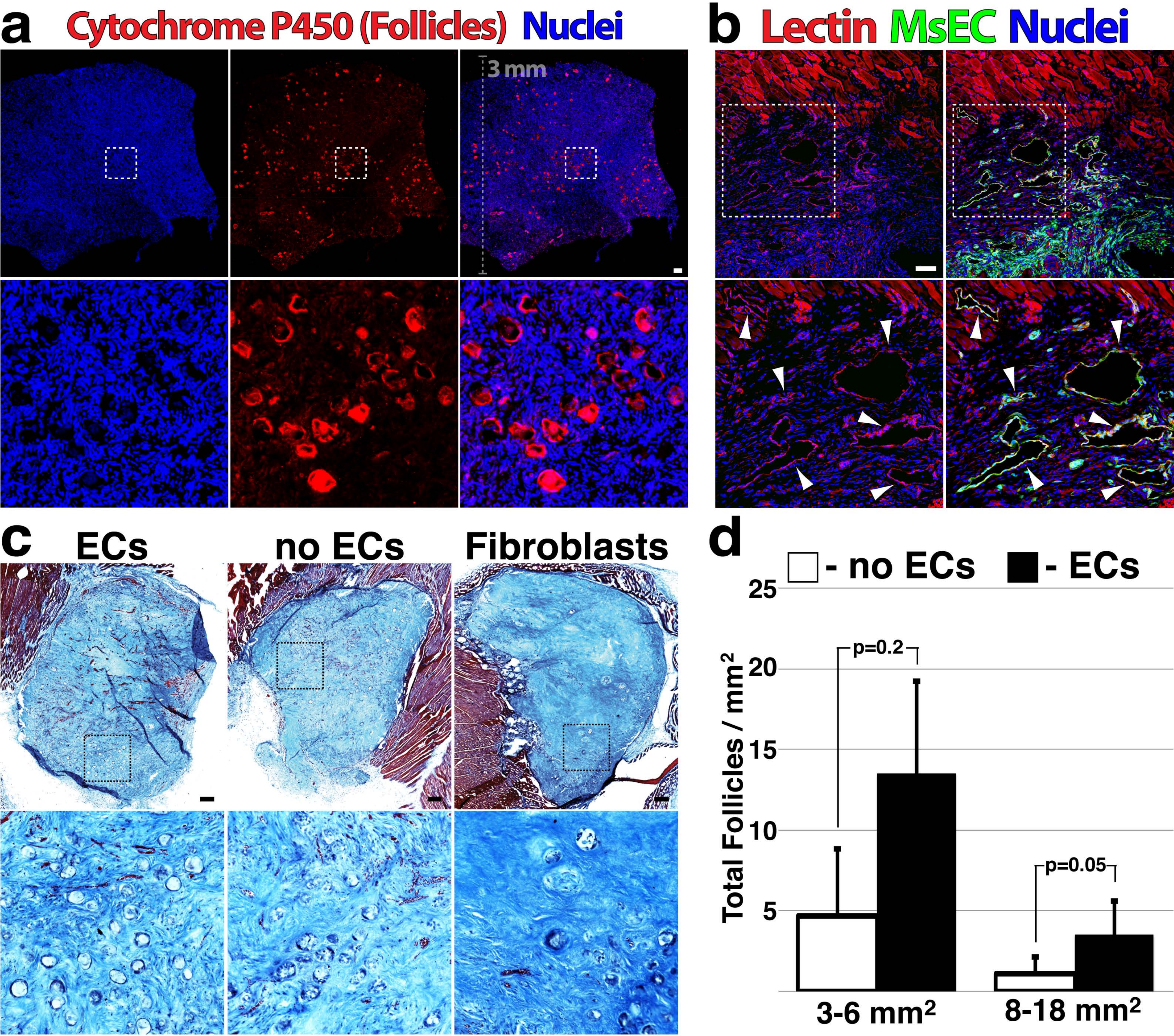

Supplement: Supplementary file 1 — Supplementary Material [file 41598_2017_8491_MOESM1_ESM.pdf]
